# Significant Correlation between High-Risk HPV DNA in Semen and Impairment of Sperm Quality in Infertile Men

**DOI:** 10.22074/ijfs.2019.5421

**Published:** 2018-10-02

**Authors:** Mansour Moghimi, Somayeh Zabihi-Mahmoodabadi, Aliasghar Kheirkhah-Vakilabad, Zahra Kargar

**Affiliations:** 1 Department of Pathology, Shahid Sadoughi Hospital, Shahid Sadoughi University of Medical Sciences, Yazd, Iran; 2 Department of Internal Medicine, Shahid Sadoughi Hospital, Shahid Sadoughi University of Medical Sciences, Yazd, Iran

**Keywords:** Human Papillomavirus, Male Infertility, Sperm Motility

## Abstract

**Background:**

Human papillomavirus (HPV) is a DNA virus that causes sexually transmitted infections (STI). Recent reports suggest that HPV may affect sperm parameters and lead to male infertility. This study aims to evaluate the cor- relation between seminal high-risk HPV infection and impairment of sperm quality in infertile Iranian men.

**Materials and Methods:**

In this case-control study, we collected fresh semen samples from 70 fertile men and 70 confirmed infertile men who referred to Yazd Infertility Centre in 2015. Semen analyses were performed according to the World Health Organization (WHO) guidelines. High-risk HPV DNA was detected by real-time polymerase chain reaction (PCR).

**Results:**

A total of 140 subjects participated in the current study. Among 70 confirmed infertile males, only 8 (11.43%) cases tested positive for high-risk HPV and all fertile men were HPV-negative. This data revealed a significant asso- ciation between high-risk HPV and male infertility (P=0.03). The percentage of normal sperm morphology and sperm motility rate significantly declined in men infected with HPV (P<0.001).

**Conclusion:**

There was a significantly higher prevalence of high-risk HPV in infertile men than fertile men. HPV infection seemed to be a risk factor for male infertility. Additional, larger studies should be conducted to confirm the impact of HPV on male infertility.

## Introduction

Human papillomavirus (HPV) infection is caused by a DNA virus and it is considered the most common sexually transmitted disease (STD) worldwide (1). Currently, over 150 different HPV genotypes have been identified and classified as low-risk or high-risk according to oncogenic potential of the virus (2, 3). Low-risk HPVs mostly cause self-limited infections such as skin warts and are not usually associated with neoplasia (4). High-risk types of HPV are strongly linked to several cancers in both men and women (5). HPV is responsible for 630,000 new cancer cases per year worldwide and accounts for 0.8% of all cancers in men and 8.6% in women (6).

HPV may be found anywhere in the male reproductive tract such as external genitalia, epididymis, vas deferens, and urethra (7). Although HPV can infect the semen, its role as a direct cause of infertility is not clear (8).

Sexually active couples who cannot achieve pregnancy after one year are considered infertile. According to this definition, approximately 15-20% of couples are infertile. Despite the available advanced diagnostic methods, approximately 20-35% of infertile men have unexplained infertility (9). Approximately 6-10% of male infertility is due to male genital tract infections.

Evidence suggests that sexually transmitted infections (STI) such as *Treponema pallidum, Chlamydia trachomatis,* and *Neisseria gonorrhoeae* can lead to reduced fertility or infertility. Sexually transmitted viral infections that include herpes simplex virus (HSV), cytomegalovirus (CMV), and human immunodeficiency virus (HIV) also alter semen parameters and fertility.

In addition, HPV has been recently considered as an infectious agent that affects fertility (10). Several studies have reported that HPV virions can bind to the head of sperm and directly reduce its motility (11). It has been suggested that HPV infections might be linked to unexplained infertility in men. In addition, a significant correlation between seminal HPV DNA infection and lower total sperm count has also been reported (12).

The prevalence of sperm infection by HPV is reported to be 2-31% in the general male population and 10-35% in men with unexplained infertility (13). Some studies have indicated a significant relationship between seminal HPV infection and sperm quality. On the other hand, other studies have not found a significant association between abnormal sperm parameters and HPV infection in infertile men (9, 14). Therefore, the effect of HPV infection on impaired sperm quality and male infertility is debatable. A few studies have sought to determine the impact of HPV on fertility in Iran, with inconsistent results (14, 15). The current study aimed to determine the correlation between seminal high-risk HPV infection and impairment of sperm quality in infertile Iranian men in an attempt to increase information about the possible effect of HPV infection on alterations of male fertility.

## Materials and Methods

We conducted this case-control study on 70 infertile male patients and 70 confirmed fertile males who referred to Yazd Infertility Center in 2015. The Ethics Committee of Shahid Sadoughi University of Medical Sciences, Yazd, Iran approved this study (IR.SSU.MEDICINE.REC.1393.130) and all participants signed the study informed consent form. The major inclusion criterion of the case group was infertility after at least one year of unprotected sexual intercourse. The main exclusion criteria of the case group were as follows: chromosome abnormalities, azoospermia, undescended testis, and history of orchitis or varicocele. In addition, we excluded men whose spouses had histories of uterine and ovarian disorders. The control group included fertile men who had at least one child. The exclusion criterion of the control group was the presence of genital warts. The semen samples were collected and allowed to liquefy for 1 hour at 37°C. The spermogram was carried out for every specimen to determine semen parameters such as total sperm number, sperm motility, and morphology according to the World Health Organization (WHO) guidelines. Semen samples were immediately stored at -20°C until nucleic acid extraction and HPV detection.

### DNA extraction and human papillomavirus detection

The semen samples were centrifuged at 2500 rpm for 10 minutes. The supernatants were removed, and the pellets were transferred to Eppendorf tubes. DNA extraction was performed with a RIBO-prep Extraction Kit (AmpliSens, Russia) according to the manufacturer’s instructions. High-risk HPV types 16, 18, 31, 33, 35, 39, 45, 51, 52, 56, 58, and 59 were detected using the AmpliSens® HPV HCR Screen-titre-FRT kit (AmpliSens, Russia) based on multiplex real-time polymerase chain reaction (PCR). The real-time PCR assays were performed using an ABI Step One Plus system (Applied Biosystems, Foster City, CA, USA). The reaction mixture in a total volume of 25 μl contained 10 μl of extracted DNA and 15 μl of the master mix according to the kit instructions. The conditions for real-time PCR assay consisted of an initial denaturation phase at 95ºC for 15 minutes followed by 60 cycles at 95ºC for 5 seconds, annealing at 60ºC for 20 seconds, and extension at 72ºC for 15 seconds.

### Statistical analysis

General descriptive data of fertile and infertile men and the values of sperm parameters were presented as mean ± SD. Data were analysed using Stata statistical software, version 14 (StataCorp LLC, College Station, TX, USA). Univariate and multivariate logistic regression analysis were used for comparison of participant characteristics and sperm parameters between infertile and fertile men. Penalized logistic regression model with data augmentation was performed to compare the frequency of high-risk HPV DNA between infertile and fertile men (16). We compared the sperm parameters in infertile men between the HPV-positive and HPV-negative group using the independent samples t test. P<0.05 were considered statistically significant.

## Results

A total of 140 men with an average age of 32.74 ± 5.21 years participated in the study. Table 1 summarizes the characteristics and sperm parameters for the fertile and infertile men. Data analysis according to logistic regression showed no statistically significant difference in fertility between smokers and nonsmokers (P=0.42). In addition, the results in Table 1 showed no significant correlation between education, length of marriage, and male infertility (P>0.05). Sperm parameters that included counts, total motility, progressive motility, and normal morphology rate in infertile men were significantly lower than the fertile group (Table 1, P<0.05). Overall, the prevalence of high-risk HPV DNA infection in semen was 5.7%. Among 70 subjects in the case group, 8 (11.43%) men were HPV positive. None of the men in the control group had any HPV infection.

Penalized logistic regression analysis via data augmentation revealed a significant association between high-risk HPV infection and male infertility (Table 1, P=0.03). The probability of fertility in high-risk HPV-positive men was 90% less than those not infected with HPV [odds ratio (OR): 0.1, 95% confidence interval (CI): 0.01-0.82]. In the case group (infertile men), sperm motility, progressive motility and percentage of normal sperm morphology of the HPV-positive subjects showed statistically significant decreases compared with HPV-negative cases (Table 2). Sperm concentration in the infertile men infected with HPV was lower than the HPV negative group, but this difference was not significant (Table 2, P=0.41).

**Table 1:** Characteristics of participants and sperm parameters in the total study population


Characteristics/parameters		Fertile men n=70	Infertile men n=70	OR adjusted	95% CI	P value^a^

Mean age (Y ± SD)		33.61 ± 5.25	31.88 ± 5.18	1.6	1.23-2.36	0.99
Education n (%)						
	Illiterate	2 (1.4)	1 (0.7)	0.37	0.27-3.12	0.61
	Primary	6 (4.3)	4 (2.9)	0.59	0.47-1.78	0.46
	Secondary	23 (16.4)	18 (12.9)	0.71	0.39-1.13	0.37
	College	39 (27.8)	47 (33.6)	1	-	Reference
Married period (Y)		5.96 ± 4	5.73 ± 4.46	0.93	0.77-1.12	0.43
Smoking (%)		17 (24.3)	23 (32.9)	0.68	0.49-1.14	0.42
Sperm count (million/ml)		93.50 ± 37.95	59.64 ± 30.21	1.01	0.95-0.98	0.021
Sperm motility (%)		59.39 ± 8.86	31.21 ± 14.04	1.05	0.80-0.89	0.042
Sperm progressive motility (%)		37.54 ± 17.08	6.09 ± 5.20	1.06	0.64-0.82	0.016
Normal sperm morphology rate (%)		40.10 ± 9.48	14.26 ± 11.45	1.03	0.78-0.88	0.031
HPV DNA infection of semen		0	8 (11.43)	0.1	0.01-0.82	0.03^b^


Data are presented as mean ± SD or n (%). HPV; Human papillomavirus, OR; Odds ratio, CI; Confidence interval, ^a^; P value obtained by univariate and multivariate logistic regression analysis, and b; P value obtained by penalized logistic regression model via data augmentation.

**Table 2:** Comparison of sperm parameters between infertile men infected with HPV and the HPV-negative group


Sperm parameters	HPV positive	HPV negative	P value^a^

Sperm count (million/ml)	51.38 ± 29.29	60.71 ± 30.39	0.41
Sperm motility (%)	23.50 ± 13.50	32.21 ± 13.90	0.04
Sperm progressive motility (%)	0.63 ± 1.77	6.79 ± 5.08	<0.001
Normal sperm morphology rate (%)	7.13 ± 2.64	15.18 ± 11.83	<0.001


Sperm parameters are presented as mean ± SD. HPV; Human papillomavirus and ^a^; Pvalue obtained by independent samples t test.

## Discussion

The effect of HPV on sperm quality and male infertility is controversial. Results of the current study have indicated that seminal high-risk HPV in infertile men was higher than in the fertile men, and it could impair sperm quality. These data suggested a possible role for HPV in male infertility. We found an HPV infection prevalence of 11.43% in semen of men who suffered from infertility. Previous studies have also reported a prevalence of HPV in infertile men that ranged from 10 to 30% (13). The few studies conducted on infertile men in Iran have estimated the HPV prevalence to be approximately 25 to 30% (15). The difference in prevalence might be due to the use of different diagnostic tests to detect HPV.

Some STI impair semen quality by inducing epididymitis, orchitis or urethritis (12, 17). The mechanism by which HPV affects sperm quality is still poorly understood. In the current study, HPV decreased significantly total sperm motility and progressive motility. Previous studies on sperm parameters in relation to HPV infection reported conflicting findings. A few studies have reported enhanced motility and progression in HPV-exposed sperm (18, 19). Bezold et al. (12) demonstrated that HPV infection in asymptomatic men was associated with low sperm quality and changes in progressive motility. Consistent with our finding, the majority of previous studies observed reduced sperm motility in men infected with HPV (20-22). In contrast, other studies have shown no association between HPV infection and sperm quality parameters (17). A study conducted in Iran has also found a significant relationship between HPV and alteration in sperm motility (15). These contradictory results might be attributed to differences in sample size and sensitivity of HPV detection methods.

In the present study, the normal sperm morphology rate in HPV-positive cases significantly decreased compared with its rate in HPV-negative men. Several studies reported no difference in sperm morphology between HPV-infected and noninfected infertile men (17). The studies conducted in Iran also have not found a significant relationship between HPV and abnormal sperm morphology (12, 13). The relationship between HPV and its negative impact on sperm morphology remains poorly understood, but it could be due to binding of HPV to the spermatozoa head (23). Yang et al. (7) found that abnormal morphology of sperm clearly increased in HPV-infected individuals.

In this study, high-risk HPV infection did not significantly affect total sperm count in infertile men. Rintala et al. obtained similar results from infertile men infected with high-risk types of HPV (24). Unlike our result, Nasseri et al. (15) reported that HPV decreased sperm cell counts in infertile Iranian men. Several studies also reported a significant relationship between the lower total sperm count and HPV infection in semen (12, 14).

The limitation of our study included the absence of data on volume and pH of semen, which was not obtained in each semen analysis. However, most previous studies have not reported any connection between seminal HPV infection and alteration in these parameters (7, 17).

## Conclusion

Taken together, the result of the present study indicated that HPV could be a risk factor for male infertility. The prevalence of high-risk HPV in infertile men was significantly higher than fertile men. HPV decreased sperm motility and normal morphology rate. Further, larger studies would be required to confirm the impact of HPV on male infertility.
